# PIEZO1 and TRPV4, which Are Distinct Mechano-Sensors in the Osteoblastic MC3T3-E1 Cells, Modify Cell-Proliferation

**DOI:** 10.3390/ijms20194960

**Published:** 2019-10-08

**Authors:** Maki Yoneda, Hiroka Suzuki, Noriyuki Hatano, Sayumi Nakano, Yukiko Muraki, Ken Miyazawa, Shigemi Goto, Katsuhiko Muraki

**Affiliations:** 1 Laboratory of Cellular Pharmacology, School of Pharmacy, Aichi Gakuin University, 1-100 Kusumoto, Chikusa, Nagoya 464-8650, Japan; tink.yuzu.0415@gmail.com (M.Y.); hsuzuki@dpc.agu.ac.jp (H.S.); nhatano@dpc.agu.ac.jp (N.H.); 15373mt@gmail.com (S.N.); ymuraki@helen.ocn.ne.jp (Y.M.); 2 Department of Orthodontics, School of Dentistry, Aichi Gakuin University, Nagoya 464-8650, Japan; miyaken@dpc.agu.ac.jp (K.M.); shig@dpc.agu.ac.jp (S.G.)

**Keywords:** TRPV4, PIEZO1, Yoda1, MC3T3-E1 cells, mechanical stimulation, cell proliferation

## Abstract

Mechanical-loading and unloading can modify osteoblast functioning. Ca^2+^ signaling is one of the earliest events in osteoblasts to induce a mechanical stimulus, thereby demonstrating the importance of the underlying mechanical sensors for the sensation. Here, we examined the mechano-sensitive channels PIEZO1 and TRPV4 were involved in the process of mechano-sensation in the osteoblastic MC3T3-E1 cells. The analysis of mRNA expression revealed a high expression of *Piezo1* and *Trpv4* in these cells. We also found that a PIEZO1 agonist, Yoda1, induced Ca^2+^ response and activated cationic currents in these cells. Ca^2+^ response was elicited when mechanical stimulation (MS), with shear stress, was induced by fluid flow in the MC3T3-E1 cells. Gene knockdown of *Piezo1* in the MC3T3-E1 cells, by transfection with siPiezo1, inhibited the Yoda1-induced response, but failed to inhibit the MS-induced response. When MC3T3-E1 cells were transfected with siTrpv4, the MS-induced response was abolished and Yoda1 response was attenuated. Moreover, the MS-induced response was inhibited by a TRPV4 antagonist HC-067047 (HC). Yoda1 response was also inhibited by HC in MC3T3-E1 cells and HEK cells, expressing both PIEZO1 and TRPV4. Meanwhile, the activation of PIEZO1 and TRPV4 reduced the proliferation of MC3T3-E1, which was reversed by knockdown of PIEZO1, and TRPV4, respectively. In conclusion, TRPV4 and PIEZO1 are distinct mechano-sensors in the MC3T3-E1 cells. However, PIEZO1 and TRPV4 modify the proliferation of these cells, implying that PIEZO1 and TRPV4 may be functional in the osteoblastic mechano-transduction. Notably, it is also found that Yoda1 can induce TRPV4-dependent Ca^2+^ response, when both PIEZO1 and TRPV4 are highly expressed.

## 1. Introduction

The bone is a dynamic tissue that undergoes constant remodeling via two well-coordinated processes: Bone formation and resorption. Two types of cells, the bone-forming osteoblasts and the bone-resorbing osteoclasts, are involved in this remodeling [1]. The osteoblasts regulate bone-formation and tissue-mineralization through the secretion of bone matrix components and produce the essential factors for the differentiation of osteoclasts. Therefore, both osteoblasts and osteoclasts are important for bone homeostasis. Mechanical loading, such as strain, compression, and fluid shear is an obligatory factor that regulates the function of osteoblasts and osteoclasts [2,3]. In fact, bone mass is upregulated by mechanical loading. Whereas, bone loss or osteoporosis is governed by unloading [4,5]. Therefore, the molecular mechanism underlying this intricate process of mechano-sensation is an important topic in the field of bone biology. In vitro studies have demonstrated that mechanical strain and shear stress by fluid flow induces an elevation of intracellular Ca^2+^ ([Ca^2+^]_i_) levels in the osteoblasts through the activation of Ca^2+^ permeable mechano-sensitive channels [6] and this elevation, in turn, changes gene expression, in order to promote osteoblast differentiation. In addition, the autocrine-acting transmitters, such as ATP and glutamate have an important role in the elevation of [Ca^2+^]_i_ levels after activation of the mechano-sensitive channels [7,8].

Among the various known mechano-sensitive channels, the importance of transient receptor potential (TRP) channels is extensively studied and the activation of TRPV4 and TRPM3 by the hypotonic solution has been shown, which induces Ca^2+^ response in osteoblastic MC3T3-E1 cells [9]. Although, the expression of other potential mechano-sensitive TRP channels, such as TRPV2, TRPM4, and TRPM7, is detected in the human and mouse osteoblasts [10,11]. It has not been clarified whether these channels are activated by the mechanical stimulations applied to the osteoblasts. On the other hand, the PIEZO family of Ca^2+^ permeable cation channels, including two isoforms, PIEZO1 and PIEZO2, have been recently identified [12]. Both PIEZO1 and PIEZO2 are directly activated by the mechanical stimulations acting on the cell membrane [13]. PIEZO1 is also activated by a chemical agonistic compound, 2-[5-[[(2,6-Dichlorophenyl)methyl]thio]-1,3,4-thiadiazol-2-yl]-pyrazine (Yoda1) [14]. PIEZO1 has been shown to be involved in red blood cell function, as the mutations in this gene resulted in dehydrated hereditary xerocytosis [15]. Moreover, it has been shown that PIEZO1 aids in integrating the vascular architecture with physiological force [16]. On the other hand, PIEZO2 is predominantly expressed in the sensory tissues. In particular, PIEZO2 is a mechano-sensor in the Merkel cells and plays a key role in mediating the moderate touch sensation in the skin [17]. In bone biology, PIEZO1 has been shown to contribute to the mechanical stress-induced osteoclastogenesis in the periodontal ligament cells of humans [18]. Moreover, PIEZO1 and TRPV4 are distinct mechano-sensors in the chondrocytes depending on the stimuli [19]: Membrane-stretch, and cell-deflection, respectively. Although, the expression and function of PIEZO1 have been previously shown in the osteoblasts [20], the information is limited, and in particular, its involvement in the mechano-sensing of shear stress, induced by fluid flow, has not been determined. However, it has been recently shown that PIEZO1 plays an important role in bone formation in mouse osteoblasts [21]. Because the expression of *Piezo1* is upregulated by mechanical stimulation (MS), PIEZO1 is an essential mechano-sensor in bone cells, as TRPV4 is described in [21,22].

In the present study, we examined the possibility that PIEZO channels may be involved in the mechano-sensation of shear stress, induced by fluid flow in the osteoblastic MC3T3-E1 cells. Because TRPV4 is commonly known as a potential mechanical sensor in bone cells [6,23]. We also tested the involvement of TRPV4 in mechano-sensation in MC3T3-E1 cells. By employing pharmacological agonists and antagonists against PIEZO1 and TRPV4, as well as siRNA technique, we demonstrated that both, PIEZO1 and TRPV4 are functionally expressed in the MC3T3-E1 cells, but only TRPV4 is essential for the mechano-sensation of MS, with shear stress upon induction by fluid flow. Moreover, the MS-induced response was inhibited by a TRPV4 antagonist HC-067047 (HC). On the other hand, Yoda1 response was also inhibited by HC in MC3T3-E1 cells and HEK cells expressing both PIEZO1 and TRPV4, while not in HEK cells only with PIEZO1. In addition, we showed that PIEZO1 and TRPV4 activation reduce the proliferation of the osteoblastic MC3T3-E1 cells.

## 2. Results

### 2.1. PIEZO1 Activation by Yoda1 in MC3T3-E1 Cells

**Figure 1:**
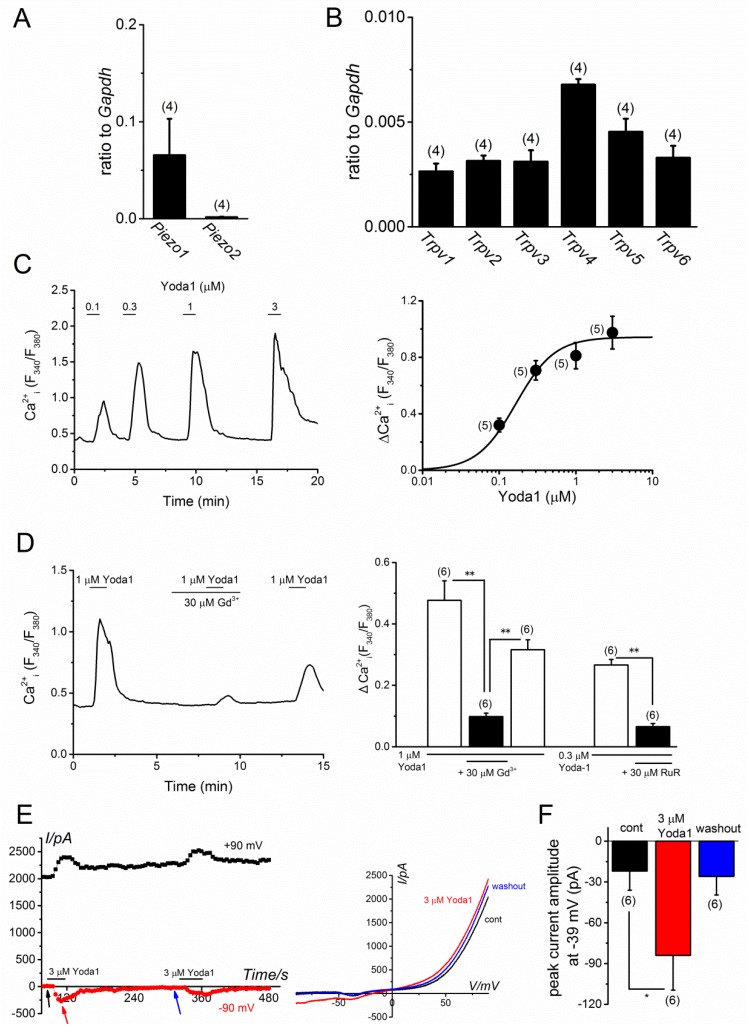
PIEZO channel expression and effects of Yoda1 in MC3T3-E1 cells. (**A**,**B**) The mRNA expression of *Piezo1* and *Piezo2* (**A**), and *Trpv1-Trpv6* (**B**) was determined in MC3T3-E1 cells with quantitative RT-PCR (four independent experiments). Each expression was shown as relative of housekeeping control, *Gapdh*. (**C**) A representative Ca^2+^ response of MC3T3-E1 cells to Yoda1 at a concentration ranging from 0.1 to 3 μM (left panel). The peak change in Ca^2+^ response of MC3T3-E1 cells to Yoda1 (ΔCa^2+^_i_ (F_340_/F_380_)) was summarized and a set of data was fitted with a concentration-response relationship (EC_50_ = 0.16 μM, *n* = 5, right panel). (**D**) A representative Ca^2+^ response of MC3T3-E1 cells to Yoda1 (1 μM) in the presence and absence of 30 μM Gd^3+^ (left panel). A summary of the peak evoked Ca^2+^ response of MC3T3-E1 cells in the presence and absence of 30 μM Gd^3+^ to Yoda1 (1 μM) and 30 μM RuR to Yoda1 (0.3 μM), respectively (right panel). (**E**) Yoda1-induced cation currents in MC3T3-E1 cells. Each cell was voltage-clamped under the whole-cell condition and treated with 3 μM Yoda1. Left panel: Ramp waveform pulses from −110 to +90 mV for 400 ms were applied every 5 s and the peak amplitude of cation currents at −90 (red line) and +90 mV (black line) were plotted against time. The arrows denote the time at which each I-V was detected. Right panel: Typical I-Vs exhibited before (a black arrow in the left) and during application of 3 μM Yoda1 (a red arrow) and after the washout of Yoda1 (a blue arrow). (**F**) A summary of the peak amplitudes of cation currents at −39 mV before, during, and after the washout of 3 μM Yoda1. The pooled data were averaged and expressed as mean ± SEM. Statistical significance was established using Student’s *t*-test and Tukey-Kramer test. * *p* < 0.05 and ** *p* < 0.01, when compared to each corresponding control group. The numbers in parentheses indicate the number of independent experiments.

To examine mouse *Piezo1* and *Piezo2* mRNA expression in the MC3T3-E1 cells, quantitative RT-PCR experiments were performed, as shown in Figure 1A. The expression of *Piezo1* and *Piezo2* was detected, and it was found that the expression level of *Piezo1* was relatively higher than that of *Piezo2*. The expression levels of each Trpv (Trpv1-v6) of the TRP super family were also confirmed because TRPV2 and TRPV4, the potential mechano-sensors, are expressed in MC3T3-E1 cells [10]. Consistently, *Trpv4* mRNA transcripts were obvious in the present study (Figure 1B). Because a chemical compound 2-[5-[[(2,6-Dichlorophenyl)methyl]thio]-1,3,4-thiadiazol-2-yl]-pyrazine (Yoda1) is known as an effective agonist against mouse and human PIEZO1 [14], Yoda1 was cumulatively applied to the MC3T3-E1 cells to test the functional expression of PIEZO1 in MC3T3-E1 cells (Figure 1C–F). The application of Yoda1 at a concentration ranging from 0.1 to 3 μM elicited a clear and reversible enhancement of intracellular Ca^2+^ levels (left, Figure 1C), and a concentration-response relationship constructed showed an effective concentration required for 50 % response (EC_50_) was 0.16 ± 0.04 μM (*n* = 5, right, Figure 1C). In addition, these Yoda1 responses were effectively inhibited by the application of Gd^3+^ and ruthenium red (RuR), non-selective cation channel blockers (Figure 1D). Next, we applied Yoda1 to MC3T3-E1 cells, which were voltage-clamped in a whole-cell clamp mode. As shown in Figure 1E, the application of 3 μM Yoda1 reversibly elicited inward and outward currents at −90 mV, and +90 mV, respectively. A current and voltage relationship (I–V) of the currents evoked, had a reversal potential of 0 mV (right, Figure 1E). To exclude the possibility of contamination of Cl^-^ currents in the Yoda1-induced currents, the current amplitudes before, and during, the application of 3 μM Yoda1 and after the washout were measured at −39 mV, where Cl^−^ currents were negligible because of the equilibrium potential of Cl^−^ (Figure 1F). It was found that Yoda1 significantly induced inward currents at this potential. Taken together, osteoblastic MC3T3-E1 cells predominantly expressed PIEZO1 and Yoda1 effectively induced a PIEZO1-dependent response. 

**Figure 2:**
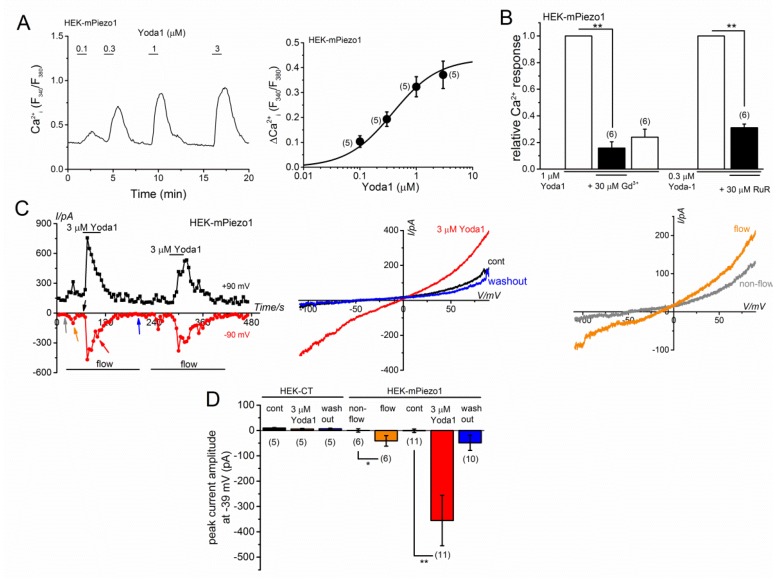
Yoda1-induced response in HEK-mPiezo1 cells. (**A**) A representative Ca^2+^ response of HEK-mPiezo1 cells to Yoda1 at a concentration ranging from 0.1 to 3 μM (left panel). The peak change in Ca^2+^ response of HEK-mPiezo1 cells to Yoda1 was summarized and a set of data was fitted with a concentration-response relationship (EC_50_: 0.38 μM, n=5, right panel). (**B**) A summary of the peak evoked Ca^2+^ response of HEK-mPiezo1 cells in the presence and absence of 30 μM Gd^3+^ to Yoda1 (1 μM) and 30 μM RuR to Yoda1 (0.3 μM), respectively (right panel). As an expression level of PIEZO1 was different from cell to cell, each Yoda1-induced response was normalized by the first. (**C**) Yoda1-induced cation currents in HEK-mPiezo1 cells. Each cell was voltage-clamped under the whole-cell condition and treated with 3 μM Yoda1. Left panel: Ramp waveform pulses from −110 to +90 mV for 400 ms were applied every 5 s and the peak amplitude of cation currents, at −90 (red line) and +90 mV (black line), was plotted against time. Arrows denotes the time at which each I-V was detected. Middle panel: I-Vs exhibited before (a black arrow) and during application of 3 μM Yoda1 (a red arrow) and after the washout of Yoda1 (a blue arrow) . Right panel: I-Vs before (non-flow, a grey arrow) and after (flow, an orange arrow) the start of fluid flow were illustrated. (**D**) A summary of the peak amplitude of cation currents at −39 mV before, during, and after the washout of 3 μM Yoda1 in HEK-CT and HEK-mPiezo1 cells. The peak amplitude of the cation currents at −39 mV before, and after, the start of fluid flow, were also summarized in HEK-mPiezo1 cells. The pooled data were averaged and expressed as mean ± SEM. Statistical significance was established using Student’s *t*-test. The * *p* < 0.05 and ** *p* < 0.01, when compared to each corresponding control group. The numbers in parentheses indicate the number of independent experiments. The mRNA expression of mouse *Piezo1* and human *PIEZO1* was determined in HEK-CT and HEK-mPiezo1 cells with quantitative PCR (Figure S2A).

To compare the response of recombinant mouse PIEZO1 to Yoda1 with MC3T3-E1 cells, we applied Yoda1 to control HEK (HEK-CT, Figure S1B) and the HEK cells transfected with mouse *Piezo1* (HEK-mPiezo1, Figure 2). Similar to the MC3T3-E1 cells, Yoda1 induced Ca^2+^ response in the HEK-mPiezo1 cells in a concentration-dependent manner (Figure 2A) and the EC_50_ was recorded as 0.38 ± 0.07 μM (*n* = 5). In contrast, HEK-CT cells slightly expressed human *PIEZO1* (Figure S1A) and clearly had a smaller response to Yoda1 than HEK-mPiezo1 cells (Figure S1B). The response to Yoda1 was effectively inhibited by the application of Gd^3+^ and RuR (Figure 2B). On the contrary, we also applied 3 μM Yoda1 to HEK-CT and HEK-mPiezo1 cells, which were voltage-clamped in a whole-cell clamp mode. As shown in Figure 2C, Yoda1 reversibly elicited inward and outward currents at −90 mV, and +90 mV, respectively, in HEK-mPiezo1 cells (left, Figure 2C), and Yoda1-induced I-V had a reversal potential of 0 mV (middle, Figure 2C). When the current amplitudes before, and during, application of 3 μM Yoda1, and after the washout were measured at −39 mV, Yoda1 clearly induced inward currents in HEK-mPiezo1 cells, but not in the HEK-CT cells (Figure 2D). This suggests Yoda1 induced the activation of PIEZO1. Furthermore, when fluid flow started after an establishment of the whole-cell recording, PIEZO1-like currents were transiently activated in some of the HEK-mPiezo1 cells (six out of eleven cells, left and right illustrations of Figure 2C,D). These results strongly suggest that the responses of MC3T3-E1 cells to Yoda1 were responsible for the activation of PIEZO1, which was potentially sensitive to the mechanical stimulation (MS) with shear stress induced by fluid flow.

### 2.2. Effects of Mechanical Stimulation in MC3T3-E1 cells and HEK Cells with Mouse PIEZO1

**Figure 3:**
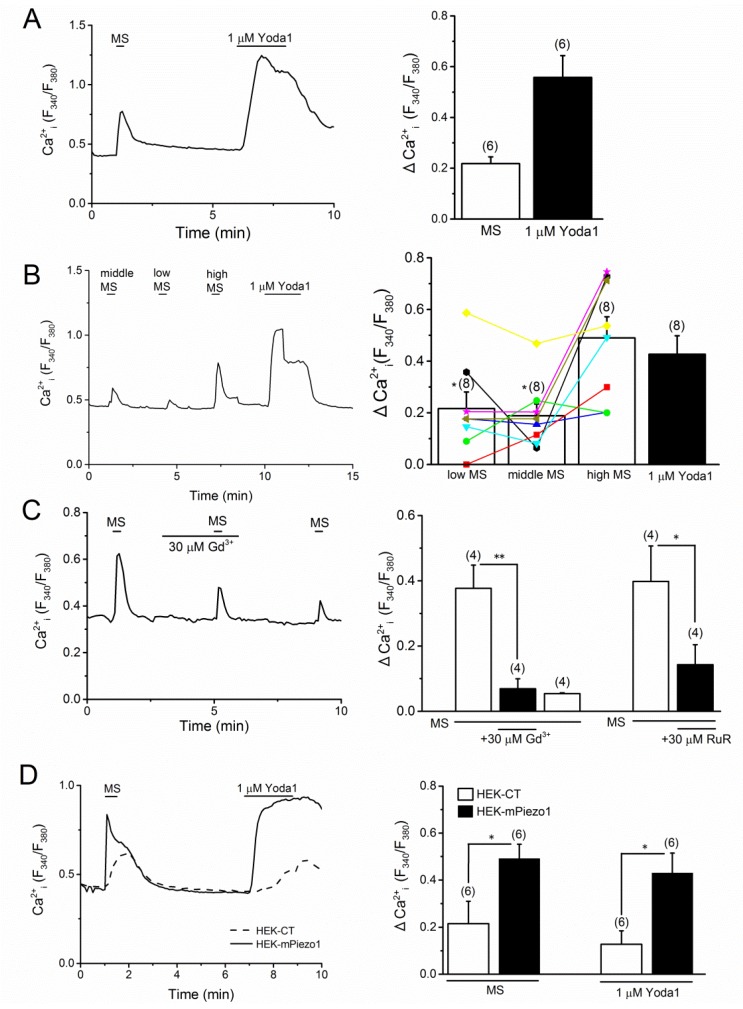
Mechanical stimulation-induced Ca^2+^ response in MC3T3-E1 and HEK-mPiezo1 cells. Mechanical stimulation (MS) with shear stress, which was induced by fluid flow, was applied to the cells monitoring Ca^2+^ response. (**A**) MC3T3-E1 cells were exposed to MS (7.67 μL/s) and 1 μM Yoda1 (left panel) and the peak change in each Ca^2+^ response was summarized as a bar chart (right panel). (**B**) MC3T3-E1 cells were exposed to MS with different shear stresses by fluid flow (middle MS: 7.67, low MS: 3.33, and high MS: 16.67 μL/s) and 1 μM Yoda1 (left), and the peak change in each Ca^2+^ response was summarized in accordance with the strength of MS (right). Different color symbols show each change of Ca^2+^ response by low, middle, and high MS in eight independent experiments. The Ca^2+^ response of a representative cell (left) was included in the averaged response in clustered cells, shown in red (right). The * *p* < 0.05 when compared to high MS group. (**C**) A representative Ca^2+^ response in MC3T3-E1 cells to MS (7.67 μL/s) in the presence, and absence, of 30 μM Gd^3+^ (left panel). A summary of the peak evoked Ca^2+^ response in MC3T3-E1 cells to MS in the presence, and absence, of 30 μM Gd^3+^ and 30 μM RuR (right panel). (**D**) HEK cells with, or without, mPIEZO1 were exposed to MS (7.67 μL/s) and 1 μM Yoda1 (left panel), and the peak change in each Ca^2+^ response was summarized as a bar chart (right panel). Pooled data were averaged and expressed as mean ± SEM. Statistical significance was established using Student’s *t*-test and Tukey-Kramer test. * *p* < 0.05 and ** *p* < 0.01, when compared to each corresponding control group. The numbers in parentheses indicate the number of independent experiments.

PIEZO1 is a putative mechanical sensor and is, thereby, activated by MS with shear stress upon induction with fluid flow, as well as membrane stretch [12,13,16]. To test the mechano-sensitivity of PIEZO1 to MS in MC3T3-E1 cells, we applied fluid flow stimulations during the measurement of Ca^2+^ response. The cells were exposed to MS through a thin tube located at a distance of 300 μm from the target cells (see Section 4). As shown in Figure 3A, MS with fluid flow at 7.67 μL/s clearly elicited a Ca^2+^ response in MC3T3-E1 cells following Yoda1 response. Systematic changes in MS (middle: 7.67, low: 3.33, and high: 16.67 μL/s) elicited Ca^2+^ responses in these cells, which were variable from stimulation to stimulation and from cell-to-cell (Figure 3B, the right panel). In addition, the MS-induced Ca^2+^ response was effectively inhibited in the presence of Gd^3+^ and RuR (Figure 3C). As shown in Figure 3D, when the MS was applied to HEK cells with, or without, mPIEZO1, a larger response to MS was elicited in the HEK-mPiezo1 cells. Consistently, the exposure to Yoda1 at 1 μM evoked a larger Ca^2+^ response in HEK-mPiezo1 cells, as compared to that in HEK-CT cells, suggesting that mPIEZO1 is sensitive to the MS produced by the flow-induced sheer stress, and hence, mPIEZO1 is a potential mechano-sensor in MC3T3-E1 cells.

### 2.3. Knockdown of PIEZO1 in MC3T3-E1 Cells

**Figure 4:**
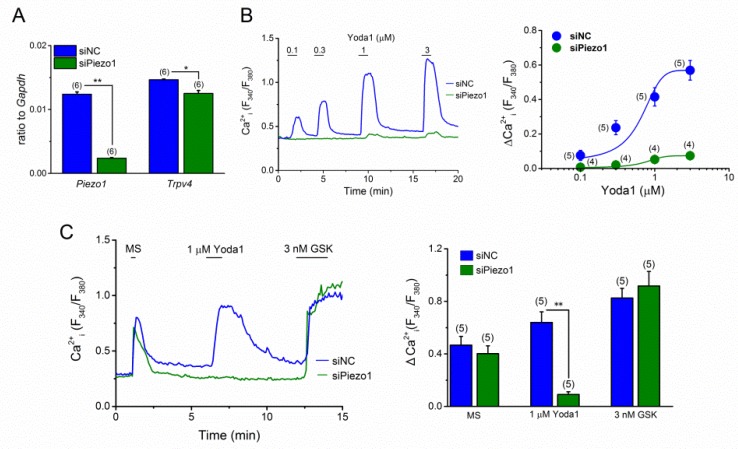
PIEZO1-knockdown in MC3T3-E1 cells and its effect on Yoda1- and MS-induced Ca^2+^ response. The expression of PIEZO1 in MC3T3-E1 cells was reduced by siPiezo1 treatment for 96 h. (**A**) The mRNA expression level of *Piezo1* and *Trpv4* in MC3T3-E1 cells transfected with siPiezo1 compared to the cells transfected with stealth control siRNA (siNC). (**B**) A representative Ca^2+^ response to Yoda1 at a concentration ranging from 0.1 to 3 μM in MC3T3-E1 cells treated with either siPiezo1 (green) or siNC (blue) (left panel). A concentration-response relationship of the peak Ca^2+^ change (ΔCa^2+^) in the cells, treated with either siPiezo1 or siNC, was summarized (right panel). (**C**) The effect of PIEZO1 knockdown on MS-induced Ca^2+^ response. A representative Ca^2+^ response to MS (7.67 μL/s), Yoda1 (1 μM), and GSK (3 nM) in MC3T3-E1 cells, treated with either siPiezo1 or siNC was shown (left panel) and summarized (right panel). Pooled data were averaged and expressed as mean ± SEM. Statistical significance was established using Student’s *t*-test. * *p* < 0.05 and ** *p* < 0.01, when compared to each corresponding control group. The numbers in parentheses indicate the number of independent experiments.

To further confirm the involvement of PIEZO1 in MS-induced Ca^2+^ response, we knocked down PIEZO1 in MC3T3-E1 cells using stealth small interfering RNA (siRNA, Figure 4). The gene expression level of *Piezo1,* mRNA was significantly reduced to 20%, as compared to the control (siNC) in the MC3T3-E1 cells with siPiezo1 transfection. While, *Trpv4* expression was slightly reduced (Figure 4A). As expected, Yoda1-induced response was dramatically reduced in these MC3T3-E1 cells with siPiezo1 (Figure 4B), strongly indicating an effective knockdown of PIEZO1 in these cells. Accordingly, we applied MS and Yoda1 to these MC3T3-E1 cells. As a comparison, we also treated these cells with GSK1016790A (GSK), a potent and selective agonist of TRPV4. As shown in Figure 4C, the response to Yoda1 at 1 μM was abolished in MC3T3-E1 cells with siPiezo1, suggesting effective deletion of PIEZO1 in these cells. However, MS-induced, as well as GSK-induced responses of MC3T3-E1 cells with siPiezo1 were not different from those with siNC treatment, thereby, demonstrating that PIEZO1 is not exclusively responsible for the MS-induced Ca^2+^ response in MC3T3-E1 cells.

### 2.4. TRPV4 Activation by MS in MC3T3-E1 Cells

**Figure 5:**
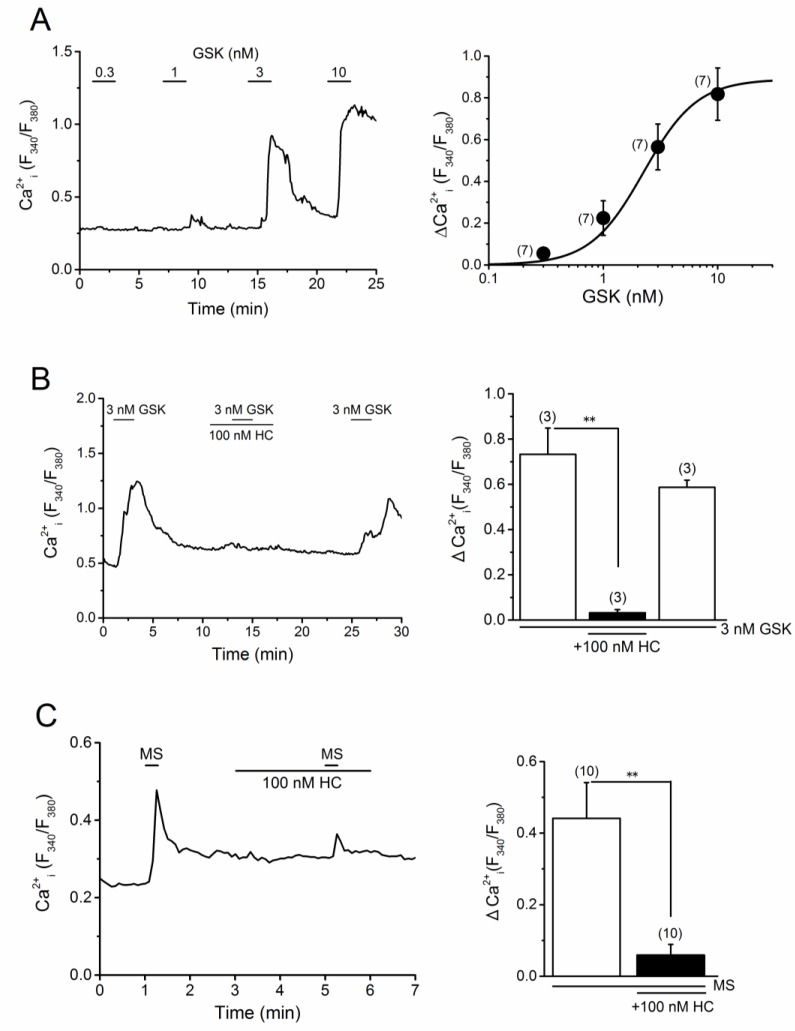
Potential involvement of TRPV4 in the MS-induced Ca^2+^ response in MC3T3-E1 cells. (**A**) A representative Ca^2+^ response of MC3T3-E1 cells to GSK, at concentrations ranging from 0.3 to 10 nM (left panel). The peak change in Ca^2+^ response to GSK was summarized in the MC3T3-E1 cells and a set of data was fitted with a concentration-response relationship (EC_50_ = 2.25 nM, *n* = 7, right panel). (**B**) A representative Ca^2+^ response of MC3T3-E1 cells to GSK (3 nM) in the presence and absence of 100 nM HC (left panel) and summary of 3 nM GSK-induced Ca^2+^ response before, during, and the washout of HC (right panel). (**C**) A representative Ca^2+^ response of MC3T3-E1 cells to MS (7.67 μL/s) in the presence and absence of 100 nM HC was shown (left panel) and summarized (right panel). Pooled data were averaged and expressed as mean ± SEM. Statistical significance was established using Student’s *t*-test. ** *p* < 0.01, when compared to each corresponding control group. The numbers in parentheses indicate the number of independent experiments.

**Figure 6:**
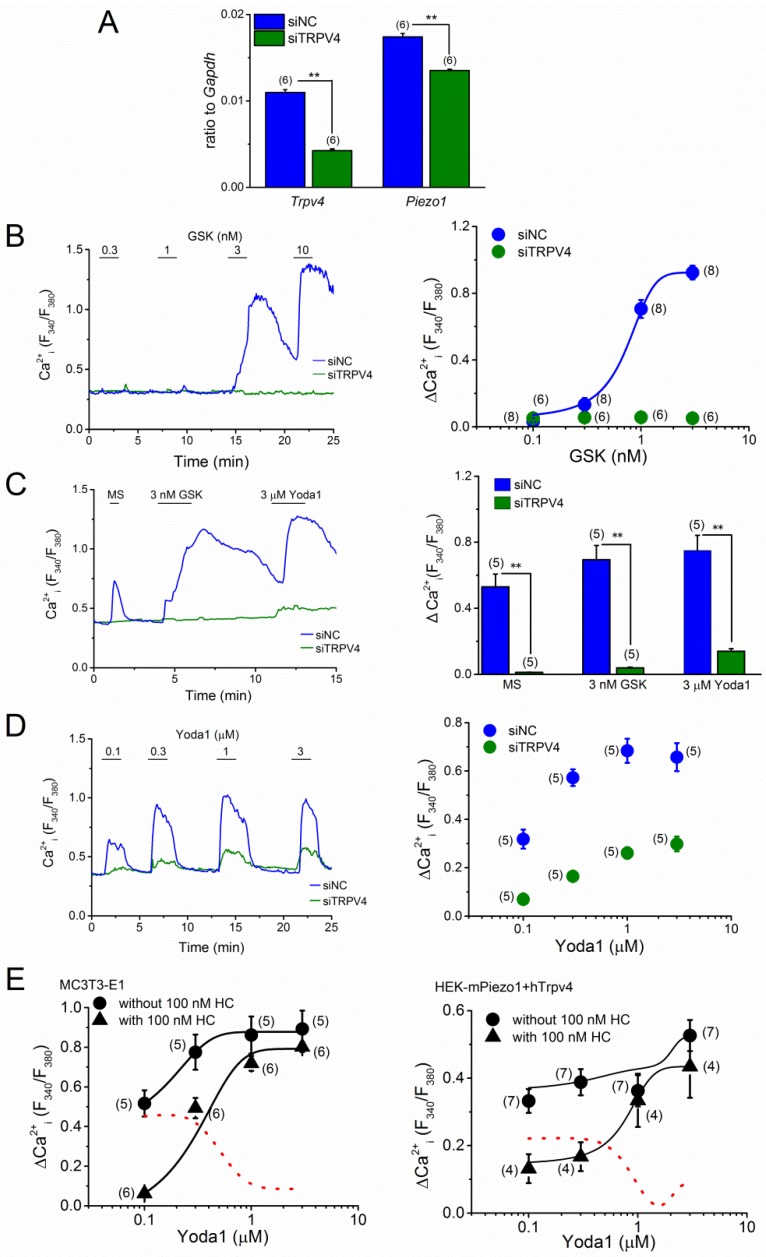
TRPV4-knockdown in the MC3T3-E1 cells and its effect on GSK- and MS-induced Ca^2+^ response. The expression of TRPV4 in MC3T3-E1 cells was reduced by siTRPV4 treatment for 96 h. (**A**) The mRNA expression level of *Trpv4* and *Piezo1* in MC3T3-E1 cells transfected with siTRPV4 compared to the cells transfected with stealth control siRNA (siNC). (**B**) A representative Ca^2+^ response to GSK at a concentration ranging from 0.3 to 10 nM (left panel) and the concentration-response relationship of GSK-induced peak change in Ca^2+^ response in the cells treated with either siTRPV4 or siNC (right panel). (**C**) The effect of TRPV4 knockdown on MS-induced Ca^2+^ response. A representative Ca^2+^ response to MS (7.67 μL/s), GSK (3 nM), and Yoda1 (3 μM) in MC3T3-E1 cells treated with either siTRPV4 or siNC was shown (left panel) and pooled data were summarized (right panel). (**D**) Concentration-response relationship of Yoda1-induced peak Ca^2+^ response in the cells treated with either siTRPV4 or siNC. A representative Ca^2+^ response change against time (left panel) and summary (right panel) was exhibited. (**E**) The effect of HC on Yoda1-induced response in MC3T3-E1 cells (left panel) and HEK cells expressing both PIEZO1 and TRPV4 (HEK-mPiezo1+hTrpv4, right panel). Each concentration-response relationship of Yoda1 was obtained in the absence, and presence of, 100 nM HC and a set of data was fitted with a concentration-response relationship. The red line shows an HC-sensitive component, which was obtained by the subtraction between curves with, or without, HC. In addition, the selectivity of Yoda1, GSK, and HC against TRPV4, PIEZO1, and Yoda1-induced PIEZO1 response, respectively, was shown in Figure S2. Pooled data were averaged and expressed as mean ± SEM. Statistical significance was established using Student’s *t*-test. ** *p* < 0.01, when compared to each corresponding control group. The numbers in parentheses indicate the number of independent experiments.

It is likely that a potential mechanical sensor TRPV4 is rather sensitive to MS in the MC3T3-E1 cells. As TRPV4 is activated by treatment with GSK, and this activation is effectively inhibited in the presence of HC-067047 (HC), a potent TRPV4 antagonist, we examined the susceptibility of MC3T3-E1 cells to both GSK and HC. As shown in Figure 5A, GSK induced a Ca^2+^ response in the MC3T3-E1 cells in a concentration-dependent manner (EC_50_ = 2.25 ± 0.45 nM) and the response to 3 nM GSK was effectively and reversibly inhibited by the addition of 100 nM of HC (Figure 5B). Accordingly, we applied MS to MC3T3-E1 cells with, or without, HC. HC (100 nM) significantly inhibited the MS-induced response, suggesting that MS can activate TRPV4 in these cells. To further confirm the involvement of TRPV4 in MS-induced response, we knocked down TRPV4 in the MC3T3-E1 cells with siTRPV4 (Figure 6). Although, the expression level of *Piezo1* mRNA in MC3T3-E1 cells with siTRPV4 transfection was slightly reduced, as shown in Figure 6A, *Trpv4* expression in these cells with siTRPV4, was clearly reduced to ~40% as compared to the control (siNC). To use these cells, we cumulatively applied GSK and confirmed an effective knockdown of TRPV4 (Figure 6B). Accordingly, we applied MS, GSK, and Yoda1 to these MC3T3-E1 cells. As shown in Figure 6C, the response to MS, as well as GSK, was clearly abolished, suggesting TRPV4 might be responsible for an MS-induced response. However, Yoda1 response of MC3T3-E1 cells with siTRPV4 was also dramatically reduced. Therefore, we further tested the effect of TRPV4 knockdown on a concentration-response relationship of Yoda1 (Figure 6D) and found that the deletion of TRPV4 reduced the Yoda1 response. The *Piezo1* mRNA expression of MC3T3-E1 cells without TRPV4 was ~80% of the control (Figure 6A), suggesting that TRPV4 may modify the Yoda1-induced response. Therefore, we next examined the effects of HC on Yoda1-induced Ca^2+^ response (Figure 6E). Pre-treatment of MC3T3-E1 cells, with 100 nM HC, reduced Yoda1-induced responses, particularly at lower concentrations of Yoda1 (left panel in Figure 6E). Consistently, pre-treatment with HC made Yoda1-induced responses of HEK cells, with heterologous expression of PIEZO1 and TRPV4, smaller (right panel in Figure 6E), suggesting that Yoda1-induced Ca^2+^ response may include a component of TRPV4 (each red line shown in Figure 6E). In contrast, Yoda1 had no effects on TRPV4 which was heterologously expressed in HEK cells (Supplementary Figure S2A). Furthermore, compared with HEK-CT cells, GSK did not induce any clear responses in HEK-mPiezo1 cells (Figure S2B), indicating that GSK did not activate PIEZO1 and endogenous expression level of TRPV4 was low in HEK cells. Because HC had no inhibitory effects on Yoda1-induced response in HEK-mPiezo1 cells (Figure S2C), endogenous TRPV4 may be not sufficient for TRPV4-dependent Yoda1 response. Taken together, in MC3T3-E1 cells where both PIEZO1 and TRPV4 are highly expressed, Yoda1 can induce TRPV4-dependent Ca^2+^ response via activation of PIEZO1.

### 2.5. Effects of PIEZO1 and TRPV4 on Proliferation of MC3T3-E1 Cells

**Figure 7:**
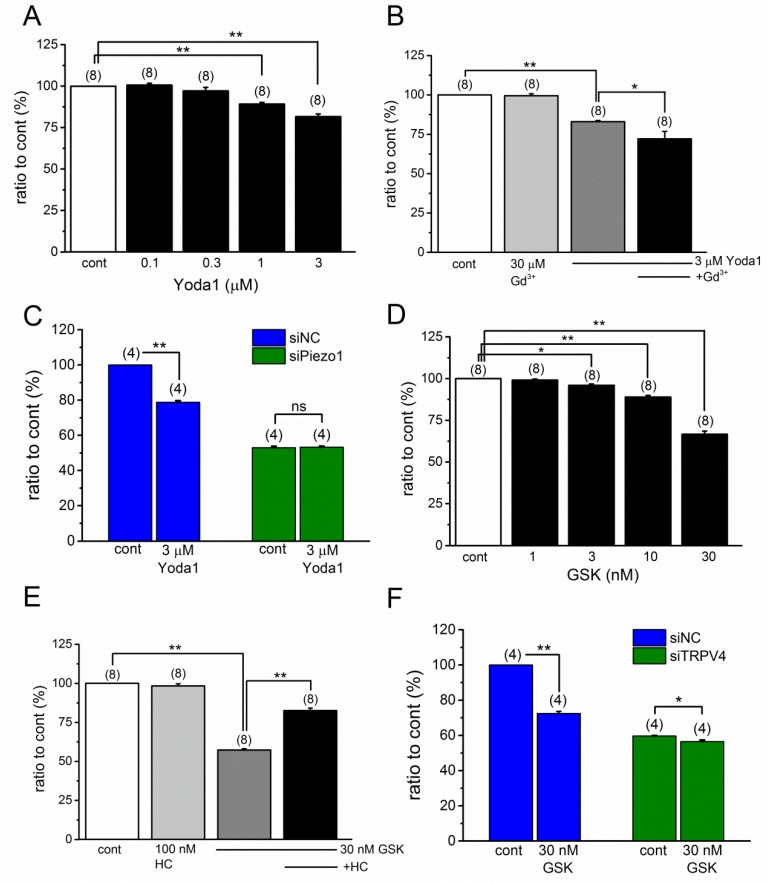
MC3T3-E1 proliferation with, or without, Yoda1 and GSK. (**A**–**F**) MC3T3-E1 cells were treated with Yoda1 (**A**) or GSK (**D**) for 24 h at a concentration ranging between 0.01 to 3 μM, and 1 to 30 nM, respectively, and cell proliferation at each concentration was measured with WST-1 assay. (**B**,**E**) Yoda1 (3 μM) or GSK (30 nM) was applied to MC3T3-E1 cells in the presence, and absence of, 30 μM Gd^3+^ (B), or 100 nM HC (E), respectively, and the cell proliferation under each experimental condition was summarized. (**C**,**F**) Yoda1 (3 μM) or GSK (30 nM) was applied to MC3T3-E1 cells transfected with siPiezo1 and siNC (**C**) or with siTRPV4, and siNC (**F**), respectively, and cell proliferation under each experimental condition was summarized. MC3T3-E1 cells were treated with siNC, siPiezo1, and siTRPV4 for 96 h. Pooled data were averaged and expressed as mean ± SEM. Statistical significance was established using Student’s *t*-test and Tukey-Kramer test. * *p* < 0.05 and ** *p* < 0.01, when compared to each corresponding control group. ‘ns’ indicates no significance. The numbers in parentheses indicate the number of independent experiments.

In our previous study, the activation of TRPV4 potentiated the proliferation of human brain capillary endothelial cells [24]. In contrast, PIEZO1 knockdown reduced the proliferation of human synovial sarcoma SW982 cells [25]. Because PIEZO1 and TRPV4 are functionally expressed in MC3T3-E1 cells, we finally examined the effects of PIEZO1 and TRPV4 on proliferation of MC3T3-E1 cells. As shown in Figure 7A, the application of Yoda1, in part, reduced the cell proliferation in a concentration-dependent manner, but the reduction was not reversed by the presence of Gd^3+^ (Figure 7B). To further confirm the potential involvement of PIEZO1 in Yoda1-induced cell proliferation reduction, Yoda1 was applied to MC3T3-E1 cells without PIEZO1 by transfection with siPiezo1 (Figure 7C). Although, PIEZO1 knockdown reduced the cell proliferation to 50%, as compared to the control, the knockdown abolished the Yoda1-induced reduction, suggesting that PIEZO1 activation reduced the proliferation of MC3T3-E1 cells. On the contrary, the treatment of MC3T3-E1 cells with GSK also reduced the proliferation in a concentration-dependent manner (Figure 7D). Furthermore, the presence of HC (Figure 7E), and the knockdown of TRPV4 (Figure 7F), effectively inhibited the GSK-induced reduction of cell proliferation. Taken together, PIEZO1 and TRPV4 can modify the proliferation of MC3T3-E1 cells.

## 3. Discussion

In the present study, we investigated mechano-sensors for shear stress, induced by fluid flow in the osteoblastic MC3T3-E1 cells. We demonstrated that two potential mechano-sensors, PIEZO1 and TRPV4, were functional in the MC3T3-E1 cells; and TRPV4, but not Piezo1, were sensitive to MS with shear stress upon induction with fluid flow for 5 s. When both PIEZO1 and TRPV4 were highly expressed, Yoda1 induced TRPV4-dependent Ca^2+^ response via activation of PIEZO1. Therefore, it is notable that Yoda1-induced Ca^2+^ response consists of TRPV4-dependent and TRPV4-independent components in MC3T3-E1 cells. In addition, the activation of PIEZO1 and TRPV4 effectively reduced the proliferation of these cells, suggesting that PIEZO1 and TRPV4 can modify osteoblast proliferation.

PIEZO channels, the novel stretch-activated channels, are expressed in neuronal and non-neuronal cells, including the bone. In primary articular chondrocytes, both PIEZO1 and PIEZO2, act as mechano-sensors for membrane stretch and the synergy between PIEZO1 and PIEZO2 play an important role in bone function [26]. Moreover, PIEZO1 is activated by cell deflection and membrane stretch in chondrocytes [19], suggesting that PIEZO1 may act as a functional mechanical sensor in chondrocytes. On the contrary, it has been shown that PIEZO1 is expressed in the mesenchymal stem cells, and human and mouse osteoblasts, including MC3T3-E1 cells [20]. Although, direct PIEZO1 activation by mechanical stimuli was not demonstrated in the study, it was shown that high pressure culture conditions (in the range of 0.01 to 0.03 MPa) effectively promoted osteogenesis, which was PIEZO1-dependent, thereby stating that PIEZO1 might act as a potential regulator of differentiation in osteoblasts. Moreover, Sun et al., recently demonstrated that PIEZO1 is involved in cationic currents, that are activated by direct membrane stretch in mouse osteoblasts, and is critical for bone formation [21]. Consistently, in our present study, a PIEZO1 agonist Yoda1 induced Ca^2+^ response and activated cationic currents in MC3T3-E1 cells. A knockdown of PIEZO1 by siPiezo1 transfection abolished Yoda1-induced response in the MC3T3-E1 cells, strongly suggesting that PIEZO1 is functionally expressed in these cells. However, the mechanical response to MS with shear stress induced by fluid flow, which was estimated to be 17.3 dyn/cm^2^, was not changed in the MC3T3-E1 cells without PIEZO1. These results provide the evidence to show that even though PIEZO1 is functional in MC3T3-E1 cells, it may not contribute to the mechanical response under the present experimental conditions. In fact, the MS by fluid flow in the present study was comparable to (17.3 dyn/cm^2^ vs. 12 dyn/cm^2^), but much shorter (5 s vs. 2 h) than that by Sun et al [21].

We can rule out the possibility that the MS of 17.3 dyn/cm^2^ , with shear stress induced by fluid flow (7.67 μL/s) was ineffective on the activation of mouse PIEZO1, as HEK-mPiezo1 cells had a larger response to the MS than the control HEK cells (Figure 3D). This suggests that the MS can activate mouse PIEZO1. Nevertheless, it is notable that HEK cells have endogenous human PIEZO1 (Figure S1) and it is possible that the expression of mouse PIEZO1 might change the channel property. On the other hand, the mechano-sensitivity may depend on the types of stimulus, membrane components and cell matrix, even though PIEZO1 is inherently mechano-sensitive [27]. Indeed, PIEZO1 and TRPV4, the two mechano-sensitive channels, have distinct sensitivity to mechanical stimulation, such as membrane stretch, mechanical deflection, and mechanical indentation in the chondrocytes [19], and hence, the MS in the present study could be insufficient to activate PIEZO1 in MC3T3-E1 cells. In the vascular endothelial cells of mouse mesenteric artery and human placental artery, MS with fluid flow at 20 μL/s evoked PIEZO1-like channel activity [28,29].

Mechanical loading with strain, compression, and shear by fluid flow are crucial stimuli to regulate the function of the bone cells, such as osteocytes, osteoclasts, and osteoblasts. Therefore, extensive studies have been done using these cells to identify the mechanical sensors involved and the downstream responding factors. When MS with 10–12 dyn/cm^2^ was applied to MC3T3-E1 cells, Ca^2+^ response was evoked by the activation of voltage-dependent L-type Ca^2+^ channel and/or unknown mechano-sensitive channels [30,31], and by intracellular Ca^2+^ release [32]. On the other hand, a fluid flow at 5 dyn/cm^2^ was sufficient to activate TRPV4 in the mouse primary osteoblast-enriched cells [6]. Compared to the shear stress on vascular endothelium in mice [33,34], the actual shear on osteoblastic cell body could be lower, due to the presence of pericellular matrix, which functions as a cushion surrounding the cells and dampens the mechanical loading [35]. Based on these physiological points, the MS applied to the MC3T3-E1 cells is relatively higher (17.3 dye/cm^2^) in the present study and may be sufficient to determine functional mechano-sensors on the cell membrane. While, it has been recently been shown that PIEZO1 is critical for bone formation [21], further investigation is required to understand the activation of PIEZO1 in the osteoblasts. 

It is evident that TRPV4 is directly involved in chondrocyte mechano-transduction. Indeed, a blockade of TRPV4 effectively inhibited matrix production in response to the compressive mechanical stimulation [36]. Moreover, mutations in the human *TRPV4* gene resulted in joint dysfunction [37]. On the contrary, osteoblast differentiation induced a higher expression of TRPV4, which was pivotal for Ca^2+^ oscillation by mechanical force [6]. In the present study, the use of a potent TRPV4 agonist GSK1016790A and antagonist HC-067047, and the knockdown of TRPV4 with siTRPV4 clearly revealed its functional expression in the MC3T3-E1 cells. Furthermore, the mechanical response to shear stress by fluid flow was significantly reduced in the MC3T3-E1 cells, pre-treated with HC-067047 and in TRPV4 deficient cells, which strongly suggests that TRPV4 contributes to the mechanical response. Consistently, TRPV4 deficiency inhibited fluid flow-induced Ca^2+^ oscillation in the mouse primary osteoblast-enriched bone cells [6]. However, an initial transient Ca^2+^ response was still induced by the fluid flow in TRPV4-deficient mouse, indicating that TRPV4 and other mechano-sensitive channels are activated by the fluid flow in osteoblasts. It has not been concluded yet that MC3T3-E1 cells have TRPV4-independent components in the MS-induced response. Indeed, a HC-067047-resistant component was shown when MS was applied to MC3T3-E1 cells (Figure 5C). Whereas, MS-induced response almost disappeared in MC3T3-E1 cells without TRPV4 (Figure 6C). It is notable that the knockdown of TRPV4 with siTRPV4 dramatically reduced Yoda1-induced response. Further analysis revealed that the Yoda1-induced response included a TRPV4-dependent component. Although, an off-target effect by siTRPV4 against Piezo1 may not be excluded, Yoda1 can induce TRPV4-depndent response via activation of PIEZO1. Nevertheless, under the present experimental conditions, PIEZO1 and TRPV4 have a distinct role in MS-induced response in the MC3T3-E1 cells, and may mediate PIEZO1- and TRPV4-dependent mechanical transduction within the bone in response to applied forces [19]. Indeed, in mouse osteoblasts PIEZO1 activation by mechanical loading enhanced the expression of alkaline phosphatase, osteocalcin, and collagen 1 at mRNA and protein levels [21]. TRPV4 activation by hypotonic stress upregulated the expression of bone remodeling factors, such as the receptor activator of nuclear factor-kappa B ligand (RANKL) and the nuclear factor of activated T cells type c1 (NFATc1) [9]. On the other hand, in mouse osteoblasts, mechanical loading and cell-differentiation promoted the expression of Piezo1, and Trpv4, respectively [6,21]. The micro-environment surrounding the osteoblasts may modify the inter-play between PIEZO1 and TRPV4 up-stream and down-stream.

It has been shown that the activation of cation channels affects cell proliferation in a positive or negative manner, depending on the channel types and/or cells. In fact, our previous study revealed that PIEZO1 knockdown reduced the proliferation of the human synovial sarcoma SW982 cells, while the activation of PIEZO1 by Yoda1 failed to change the proliferation [25]. In contrast, TRPV4 activation potentiated the proliferation of the human brain capillary endothelial cells [24]. Moreover, it has been found that PIEZO1 has important roles in the initial stages of osteoblast differentiation and bone formation, including the MC3T3-E1 cells [20,21]. In particular, PIEZO1-dependent ERK1/2 and p38 MAPK signaling cascade promotes the differentiation via induction of BMP2 expression in the osteoblasts. In the present study, PIEZO1 activation by Yoda1 effectively inhibited the proliferation of MC3T3-E1 cells. Because PIEZO1 was not responsible for MS-induced response in the MC3T3-E1 cells, in the present study, the functional relevance of Yoda1-dependent reduction in proliferation of these cells is not clear. Moreover, PIEZO1 activation by Yoda1 can induce a TRPV4-dependent response in MC3T3-E1 cells, where TRPV4 expression is relatively high. Nevertheless, the knockdown of PIEZO1 reduced cell proliferation to 50%, even without the application of Yoda1 (Figure 7C), which strongly suggests that PIEZO1 itself is critical for cell proliferation. Indeed, PIEZO1 interacts with adhesion molecules [38], and hence, the knockdown of PIEZO1 may induce the detachment of cells during cell-culture, and following the inhibition of the proliferation. It is consistent with the data showing knockdown of PIEZO1 reduced migration of MC3T3-E1 cells [39] and bone formation [21]. Therefore, even PIEZO1 activation and PIEZO1 knockdown seem to be opposite directions functionally, both possibly inhibit the proliferation of MC3T3-E1 cells. Similarly, in our study, TRPV4 activation reduced proliferation when relatively higher concentrations of GSK1016790A were applied to the MC3T3-E1 cells. Because the presence of HC-067047 and the knockdown of TRPV4 largely revered the reduction, a regulatory role of TRPV4 in the cell proliferation is clear. Similar to PIEZO1, TRPV4 *per se* is attributable to proliferation of MC3T3-E1 cells because the knockdown of TRPV4 reduced the proliferation to 60% as compared to the control (Figure 7F). Even though TRPV4 activation and TRPV4 knockdown were functionally opposite, both inhibited the proliferation of MC3T3-E1 cells. In fact, some TRPV4 mutants gain functioning (TRPV4 activation) and cause cell-death via Ca^2+^ overloading [40]. In contrast, it is not clear why TRPV4 knockdown inhibited proliferation. Because HC did not inhibit basal proliferation (Figure 7E), the basal activity of TRPV4 is not found to be important for this regulation. Alternatively, PIEZO1 may be critical for the reduction in cell proliferation in TRPV4 deficient MC3T3-E1 cells, even without Yoda1. This study does not elucidate the molecular mechanisms involved in PIEZO1- and TRPV4-dependent reduction in cell proliferation. It is well-known that Ca^2+^ overloading induces anti-proliferative activity by the activation of Ca^2+^ permeable cation channels. In contrast, the activation of Ca^2+^ permeable TRPC4 channel reduced cell proliferation in a Ca^2+^-independent manner, in the human synovial sarcoma [41]. Our future goal is to understand the mechanisms involved in PIEZO1- and TRPV4-dependent proliferation of the osteoblasts.

## 4. Methods and Materials

### 4.1. Reagents

The following reagents were used: 2-[5-[[(2,6-Dichlorophenyl)methyl]thio]-1,3,4-thiadiazol-2-yl]-pyrazine (Yoda1, Tocris Bioscience, Bristol, UK), GSK1016790 A (GSK, Sigma/Aldrich, St. Louis, MO, USA), HC-067047 (HC, Sigma/Aldrich), GdCl_3_ (Gd^3+^, Sigma/Aldrich), and ruthenium red (RuR, Sigma/Aldrich). Each reagent was dissolved in the vehicle recommended by the manufacturer.

### 4.2. Cell Culture

Mouse MC3T3-E1 (MC3T3-E1, Riken Cell Bank, Tsukuba, Japan) and human embryonic kidney 293 cell line (HEK, Health Science Research Resources Bank, Osaka, Japan) were maintained in the culture media, as recommended by the manufacturers and were used in the present experiments. All culture media were supplemented with 10% heat-inactivated FCS (GIBCO, Waltham, MA, USA), penicillin G (100 U/mL, Meiji Seika Pharma Co., Ltd., Tokyo, Japan), and streptomycin (100 μg/mL, Meiji Seika Pharma Co., Ltd.).

### 4.3. Recombinant Expression of Mouse PIEZO1 in HEK Cells

Partially confluent HEK cells (60% confluency) were transfected with the pcDNA3.1 and pIRES2-AcGFP1 plasmids containing mouse *Piezo1* (Addgene, Watertown, MA, USA) and with the pcDNA3.1 plasmid containing human *TRPV4*, using Lipofectamine 3000 (Thermo Fisher Scientific, Yokohama, Japan). The construct was verified by sequencing. The cells were used for further experiments, within 48 h after transfection.

### 4.4. Real-Time Quantitative PCR

Real-time quantitative PCR was performed using SYBR Green on a Thermal Cycler Dice Real Time System as described previously [41] (Takara Bio, Inc., Kusatsu, Japan). Transcriptional quantification of the gene products was performed relative to mouse *Gapdh* and human *GAPDH*. Each cDNA sample was tested in duplicate. The program used for quantitative PCR amplification consisted of a 30 s activation of Ex Taq™ DNA polymerase at 95 °C, a 15 s denaturation step at 95 °C, a 60 s annealing, and an extension step at 60 °C (for 45 cycles), along with a final dissociation step (15 s at 95 °C, 30 s at 60 °C, and 15 s at 95 °C). The oligonucleotide sequences of primers specific for mouse *Piezo1,* mouse *Piezo2,* mouse *Trpv1-v6,* and mouse *Gapdh* are: GCGGCGCTATGAGAACAAG (forward), CTGCGAGCGGTGGAAGA (reverse), CAGACAAGGAAGGATCGGATGA (forward), GGTCACGTGGACAGACTCTACAGA (reverse), CTTCTGAGGGACGCAAGCA (forward), CCTCAGCATCCTCTGGCTTAA (reverse), GAGGCCCGAAGTCCCAAAG (forward), CAGCTCTAGGGAGGCATCCA (reverse), GAGCCTCTGCATACGCTGCTA (forward), GAGACAAGGGTCAGGGTGATG (reverse), AACCCCATTGACCTGTTGGA (forward), GGTAAGTGCCGTAGTCGAACAAG (reverse), GACCTGCCAATTACAGAGTGGAT (forward), CAGTGAGTGTCGCCCATCAT (reverse), CCGATGAGCTGGGTCATTTC (forward), GAAGGGCAGATCCACGTCATA (reverse), and CATGGCCTTCCGTGTTCCT (forward), CCTGCTTCACCACCTTCTTGA (reverse), respectively. The oligonucleotide sequences of primers specific for human *PIEZO1* and *GAPDH* are: TAGCCATTACTACCTGCACGTC (forward), TGCGGTGAAAGTCAATGCTC (reverse) and CCTGCACCACCAACTGCTTAG (forward), TCTTCTGGGTGGCAGTGATG (reverse), respectively.

### 4.5. Voltage-Clamp Experiments

Whole-cell patch-clamp experiments were performed, as previously described [41]. The resistance of pipettes was recorded as 3–5 MΩ when filled with pipette solution. A Cs-aspartate rich pipette solution was used: 110 mM Cs-aspartate, 30 mM CsCl, 1 mM MgCl_2_, 10 mM HEPES, 1 mM EGTA, and 2 mM Na_2_ATP (adjusted to pH 7.2 with CsOH). The membrane currents and voltage signals were amplified with a EPC-800 amplifier (HEKA, Lambrechit, Germany) and digitized at 10 KHz using an analogue-digital converter (PCI6229, National Instruments Japan, Tokyo, Japan), driven by WINWCP5.2, for data acquisition and analysis for the whole-cell currents (developed by John Dempster, University of Strathclyde, Glasgow, UK). The liquid junction potential between the pipette and bath solutions was corrected (−10 mV). A ramp voltage protocol from −110 mV to +90 mV for 400 ms was applied every 5 s from a holding potential of −10 mV. A standard HEPES-buffered bathing solution (SBS: 137 mM NaCl, 5.9 mM KCl, 2.2 mM CaCl_2_, 1.2 mM MgCl_2_, 14 mM glucose, 10 mM HEPES, adjusted to pH 7.4 with NaOH) was used. Under voltage-clamp conditions, a non-laminar fluid flow with SBS at 3.8 mL/min was applied to each cover slip. All experiments were performed at 25 ± 1 °C.

### 4.6. Measurement of Ca^2+^ Fluorescence Ratio

Cells were loaded with 10 μM Fura2-AM (Dojindo, Kumamoto, Japan) in SBS for 30 min at 24–26 °C, and thereafter, superfused with SBS for 10 min to washout the Fura-2AM. Fura-2 fluorescence signals were measured every 5 s using the Argus/HisCa imaging system (Hamamatsu Photonics, Hamamatsu, Japan), driven by Imagework Bench 6.0 (INDEC Medical Systems, Santa Clara, CA, USA). For each analysis, the whole cell area was chosen as the region of interest to average the fluorescence ratio. For quantitative measurement of change in Ca^2+^ response, we collected 50 and 20 single cells on one coverslip for analysis of HEK and MC3T3-E1 cells, respectively, and repeated the same experiment with the other coverslips to reduce variation. For constructing a concentration-response curve, a set of the summarized data was fitted to a standard Hill equation (Origin J9.1, LightStone, Tokyo, Japan). To apply laminar fluid flow, cells were maneuvered to the exit of a thin capillary tube with tip diameter of 350 μm, out of which SBS flowed at 3.33, 7.67, and 16.67 μL/s for 5 s. Calculation of shear stress (τ) was done using the Hagen-Poiseuille equation (τ = 4 μQ/πR^3^), where μ is dynamic viscosity, Q is flow rate, and R is radius of the capillary tube [28,42].

### 4.7. Knockdown of PIEZO1 and TRPV4 by RNA Interference

The sequence of the stealth short interfering RNA (siRNA) duplex oligonucleotides against mouse *Piezo1* (siPiezo1, Invitrogen, Carlsbad, CA, USA) and *Trpv4* (siTRPV4, Invitrogen) is as follows: 5′-UACCGAUCUCCAGAGACCAUGAUUA-3′ for the sense strand and 5′-UAAUCAUGGUCUGUGGAGAUCGGUA-3′ for the antisense strand and 5′-CCAAGAUGUACGACCUGCUGCUUCU-3′ for the sense strand and 5′-AGAAGCAGCAGGUCGUACAUCUUGG-3′ for the antisense strand, respectively. As a negative control for the siRNA treatment, Medium GC Stealth RNAi Negative Control Duplex (siNC, Invitrogen) was used. The cells grown in a 35-mm dish or a 24-well plate were washed with fresh culture medium without antibiotics 3 h prior to transfection. The siRNA or siNC (20 µM, 5 µL for the 35-mm dish and 20 µM, 1.25 μL for the 24-well plate) and Lipofectamine RNAiMAX (2.5 µL for the 35-mm dish and 0.62 μL for the 24-well plate; Invitrogen) were diluted in 200 µL (35-mm dish) and 50 μL (24-well plate) Opti-MEM (Invitrogen), and mixed together and incubated for 20 min at room temperature for complex formation. The entire mixture was added to each well, resulting in a final concentration of 40 nM for both siRNA and siNC. The cells were incubated for 96 h in a CO_2_ chamber.

### 4.8. Cell Proliferation Assayed by WST-1

The cells were seeded onto 24-well plate 24 h prior to WST-1 measurements (2 × 10^4^ MC3T3-E1 cells per well). The cell proliferation reagent WST-1 (Roche Applied Science, Penzberg, Germany) was used in accordance with the manufacturer’s instructions. Cell proliferation, as a result of the mitochondrial dehydrogenase activity, was inferred from an increase in the yield of tetrazolium salt WST-1-induced formazan, which was determined by measuring the absorbance at 450 nm (SPARK, TECAN Japan Co. Ltd., Kawasaki, Japan). The background absorbance at the reference wavelength (620 nm) was subtracted from each reading. Each sample was tested in duplicate or triplicate and each set of the pooled data was summarized across independent experiments.

### 4.9. Statistical Analyses

Data is expressed as the mean ± SEM. Statistical significance between two groups and among multiple groups was examined using paired- or unpaired-Student’s *t*-test and Tukey Kramer test, respectively (Origin J9.1). For all tests, *p* values below 0.05 were considered statistically significant.

## 5. Conclusions

MC3T3-E1 cells have functional expression of PIEZO1 and TRPV4. The short MS with shear stress induced by fluid flow effectively activates TRPV4 but not PIEZO1. Notably, when both PIEZO1 and TRPV4 are highly expressed, a PIEZO1 agonist Yoda1 can induce TRPV4-dependent Ca^2+^ response via activation of PIEZO1. While, PIEZO1 and TRPV4 deficiency inhibit the proliferation of MC3T3-E1 cells, PIEZO1 and TRPV4 activation also reduces the cell proliferation, thereby suggesting that PIEZO1 and TRPV4 modify the proliferation of osteoblasts.

## Supplementary Materials

Supplementary materials can be found at https://www.mdpi.com/1422-0067/20/19/4960/s1.
