# Supplementary material for: PIEZO1 and TRPV4, which Are Distinct Mechano-Sensors in the Osteoblastic MC3T3-E1 Cells, Modify Cell-Proliferation
Source: Int J Mol Sci. 2019 Oct 8;20(19):4960. doi: 10.3390/ijms20194960 (PMC6801562; doi:10.3390/ijms20194960)
Supplement: Supplementary file 1 [file ijms-20-04960-s001.pdf]

*Supplementary Information*

# PIEZO1 and TRPV4, which Are Distinct Mechano-Sensors in the Osteoblastic MC3T3-E1 Cells, Modify Cell-Proliferation

**Maki Yoneda [1,2], Hiroka Suzuki [1], Noriyuki Hatano [1], Sayumi Nakano [1], Yukiko Muraki [1], Ken Miyazawa [2], Shigemi Goto [2] and Katsuhiko Muraki [1,*]**

[1] Laboratory of Cellular Pharmacology, School of Pharmacy, Aichi Gakuin University, 1-100 Kusumoto, Chikusa, Nagoya 464-8650, Japan

[2] Department of Orthodontics, School of Dentistry, Aichi Gakuin University, Nagoya 464-8650, Japan

* Correspondence: kmuraki@dpc.agu.ac.jp, Tel: +81-52-757-6788

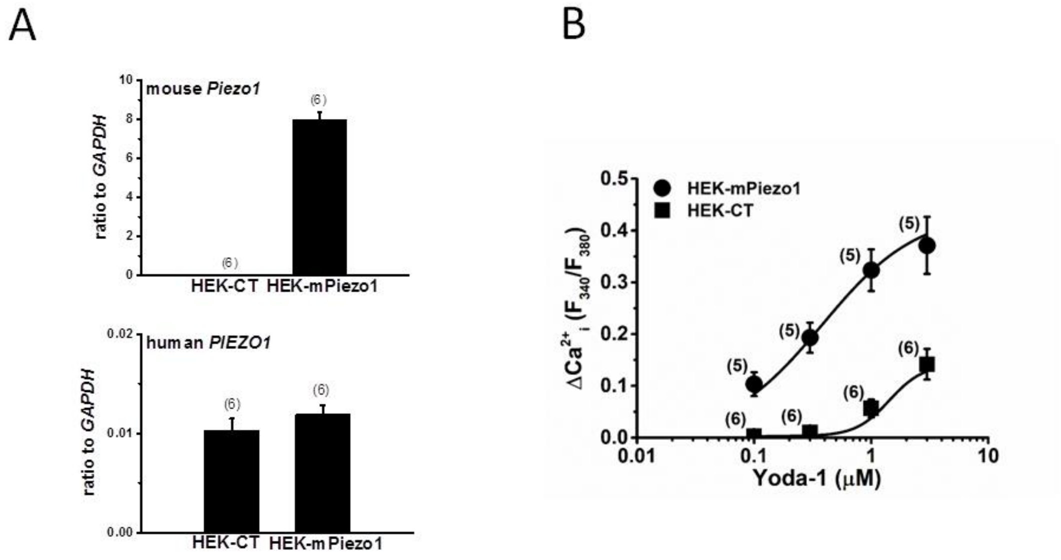


**Figure S1.** PIEZO channel expression and effects of Yoda1 on HEK-CT and HEK-mPiezo1 cells. (A) The quantitative mRNA expression of mouse *Piezo1* and human *PIEZO1* was determined in HEK-CT and HEK-mPiezo1 cells. (B) To functionally confirm the basal expression of PIEZO1 in HEK-CT cells, Yoda1 applied to HEK-CT cells. The peak change in $Ca^{2+}$ response to Yoda1 ($\Delta Ca^{2+}_i$ ($F_{340}/F_{380}$)) was summarized and a set of data was fitted with a concentration-response relationship. The same data sets in Fig.2A were used for HEK-mPiezo1 cells. Pooled data were averaged and expressed as mean ± SEM. The numbers in parentheses indicate the number of independent experiments.

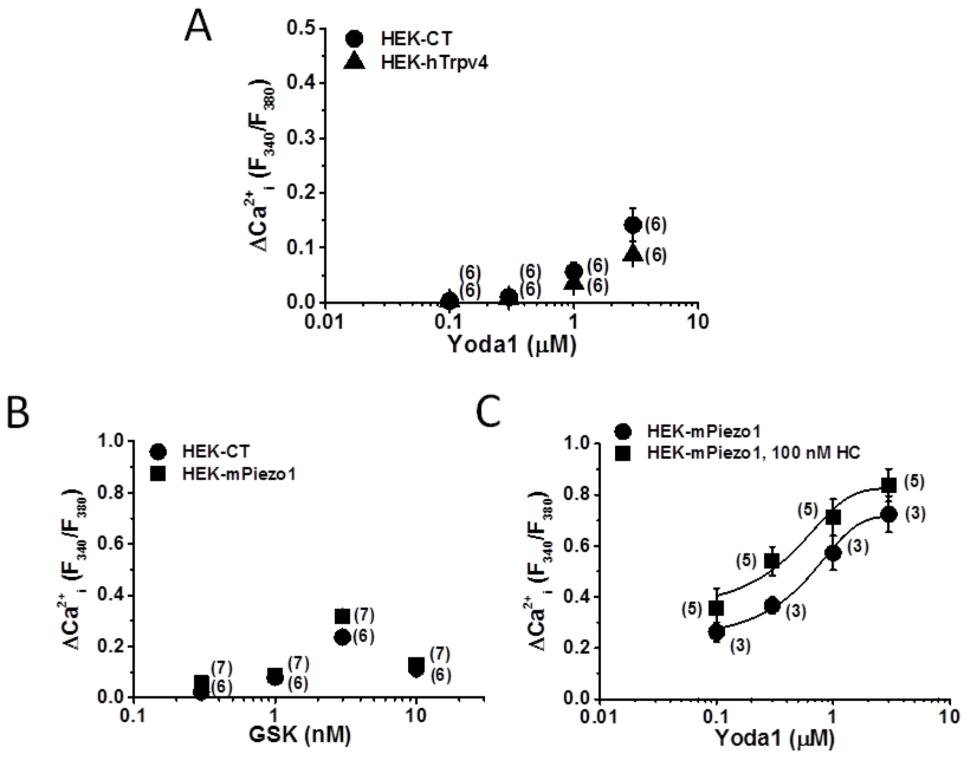


**Figure S2.** Selectivity of Yoda1, GSK, and HC against TRPV4 (A), PIEZO1 (B), and Yoda1-induced PIEZO1 response (C), respectively, was tested and the peak change in each $Ca^{2+}$ response ($\Delta Ca^{2+}_i$ ($F_{340}/F_{380}$)) was summarized as a concentration response relationship. A set of data (C) was fitted with a concentration-response relationship. Pooled data were averaged and expressed as mean ± SEM. The numbers in parentheses indicate the number of independent experiments.
